# Supplementary material for: Hyaluronic acid hydrogels support to generate integrated bone formation through endochondral ossification in vivo using mesenchymal stem cells
Source: PLoS One. 2023 Feb 2;18(2):e0281345. doi: 10.1371/journal.pone.0281345 (PMC9894498; doi:10.1371/journal.pone.0281345)
Supplement: S1 Table — (DOCX) [file pone.0281345.s001.docx]

**S1 Table. Primer sequences used in quantitative RT-PCR.**

| Gene | Primer sequences (forward and reverse, 5’–3’) |
| --- | --- |
| Aggrecan (ACAN) | TGGTGATGATCTGGCACGAG  CGTGAGCTCCGCTTCTGTAG |
| Bone sialoprotein (BSP) | TCAATCTGTGCCACTCACTGC  TTGGTGATTGCTTCCTCTGGC |
| Collagen I (Col I) | TGCAACATGGAGACTGGTGA  CCGCCATACTCGAACTGGAA |
| Collagen II (Col II) | ACAGAGGAGAAGCTGGTGC  TGCAGACCAGTGAAGCCAC |
| Collagen X (Col X) | TCCATCTGAGAATATGCTGCCA  ACCTTGCTCTCCTCTTACTGC |
| Matrix metalloproteinase13 (MMP-13) | AAGGAGCATGGCGACTTCTAC  CAAGACCTAAGGAGTGGCCG |
| Osteocalcin (OCN) | CCACCGAGACACCATGAGAG  CTCTTCACTACCTCGCTGCC |
| SOX-9 | AGGAAGTCGGTGAAGAACGG  CGCCTTGAAGATGGCGTTG |
| Tyrosine 3-monooxygenase/tryptophan 5-monooxygenase activation protein zeta (YWHAZ) | GATGAAGCCATTGCTGAACTTGA  GGTATCCGATGTCCACAATGTCA |
| GAPDH | AGGTGAAGGTCGGAGTCAAC  CCATGTAGTTGAGGTCAATGAAGG |
