# Supplementary figures and images for: Hyaluronic acid hydrogels support to generate integrated bone formation through endochondral ossification in vivo using mesenchymal stem cells
Source: PLoS One. 2023 Feb 2;18(2):e0281345. doi: 10.1371/journal.pone.0281345 (PMC9894498; doi:10.1371/journal.pone.0281345)

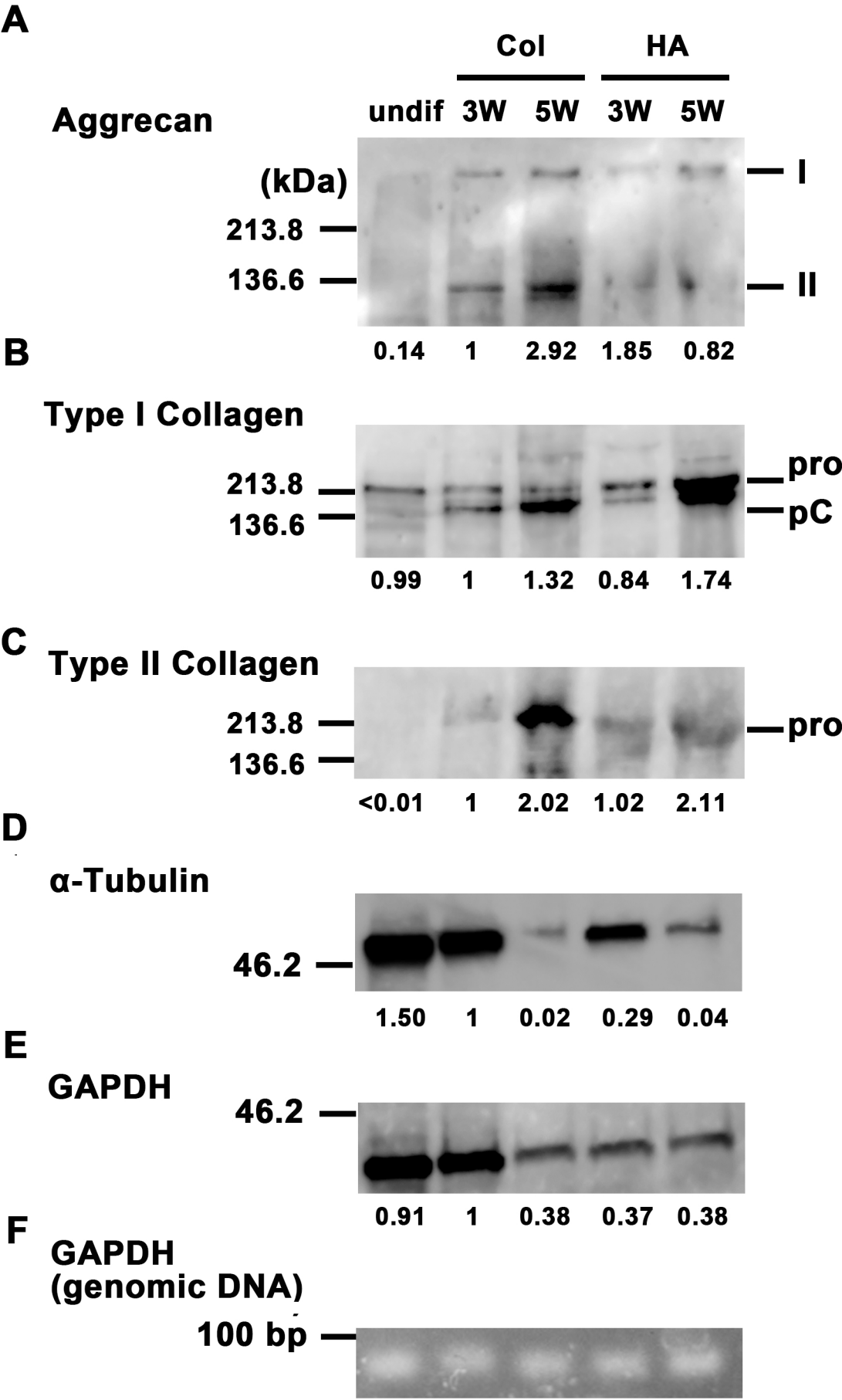

Supplement: S1 Fig — (A-C) Undifferentiated MSCs expressed Col I but no or very low levels of ACAN and Col II. Except for ACAN in the HA constructs, there was an increased accumulation of Col I, Col II, and ACAN proteins at 5W than 3W. (A) I and II: Uncleaved and cleaved fragments, respectively. (B and C) Unprocessed (pro) or processing intermediate of pro-collagen containing carboxy-pro-peptide (pC). (D and E) GAPDH and α-Tubulin were used as loading controls. The numbers were shown below the bands relative to the Col constructs at 3-week culture. (F) Representative of GAPDH PCR of genomic DNA in the samples loaded into a gel. (PDF) [file pone.0281345.s002.pdf]

**SOX-9****MMP-13****Collagen****3W**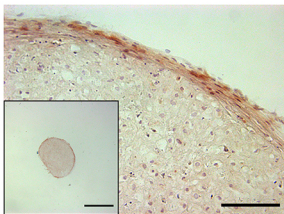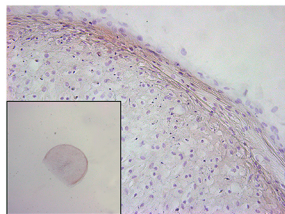**5W**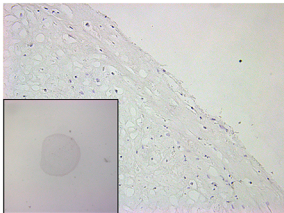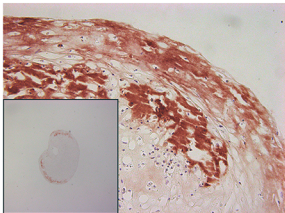**HA****3W**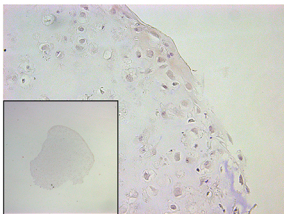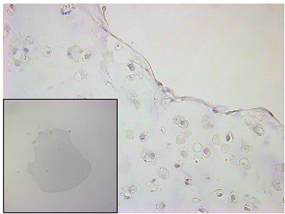**5W**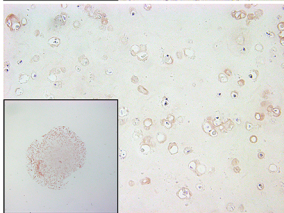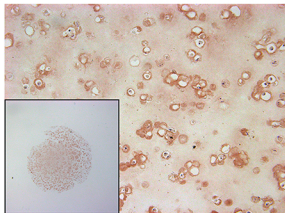

Supplement: S2 Fig — Constructs were stained for Sox-9 and MMP-13. All pictures were captured at the same magnification (Scale bar: 200 μm). A low magnification overview of the entire tissues is shown in the insets (Scale bar: 1 mm). (PDF) [file pone.0281345.s003.pdf]
