# Supplementary material for: Hyaluronic acid hydrogels support to generate integrated bone formation through endochondral ossification in vivo using mesenchymal stem cells
Source: PLoS One. 2023 Feb 2;18(2):e0281345. doi: 10.1371/journal.pone.0281345 (PMC9894498; doi:10.1371/journal.pone.0281345)
Supplement: S1 Raw images — (PDF) [file pone.0281345.s004.pdf]

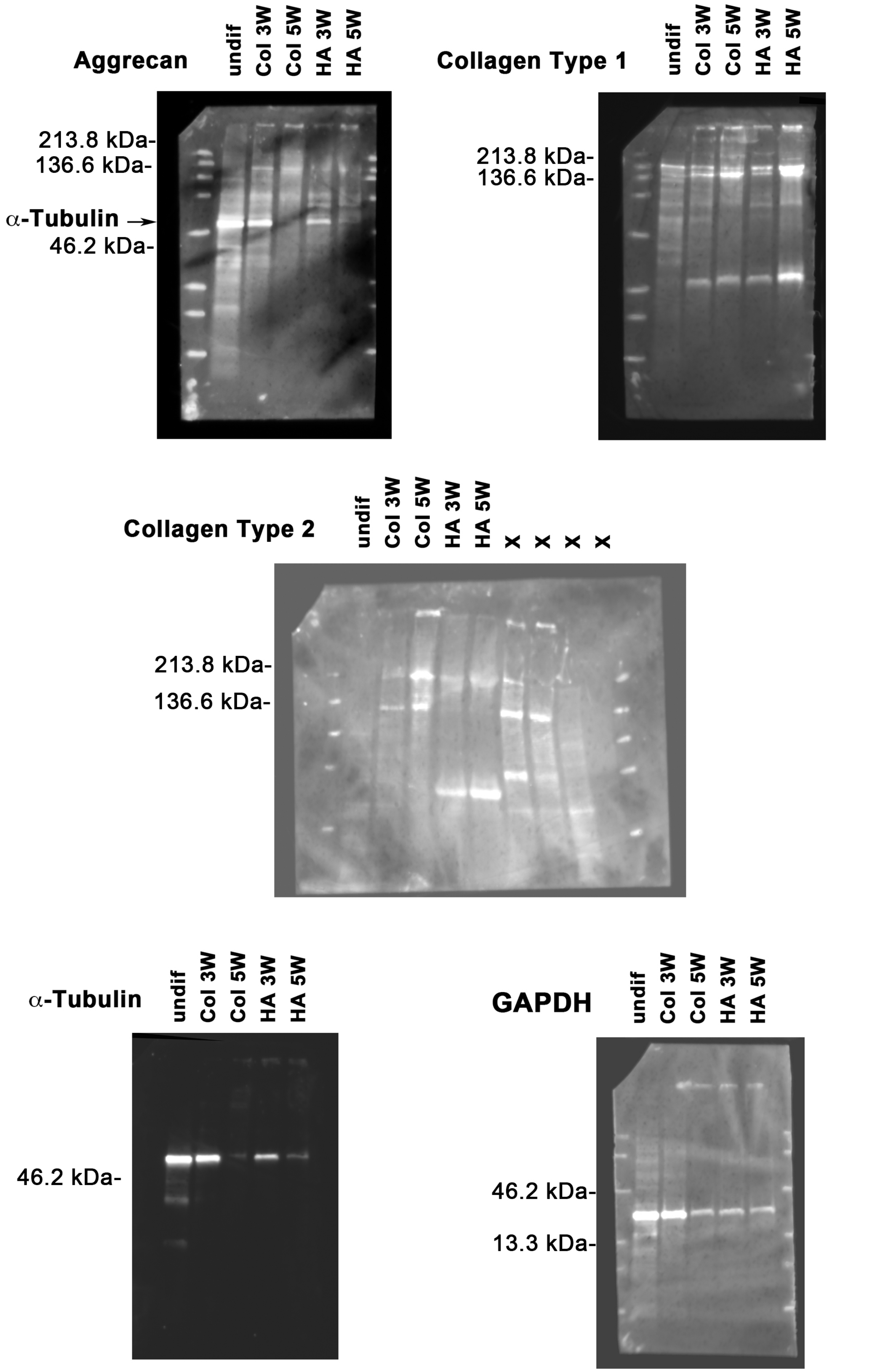

Western blot images of Aggrecan, Type I and II collagens, alpha-Tubulin, and GAPDH were digitally captured using a CCD camera after ECL treatment.
