# Supplementary material for: Hyaluronic acid hydrogels support to generate integrated bone formation through endochondral ossification in vivo using mesenchymal stem cells
Source: PLoS One. 2023 Feb 2;18(2):e0281345. doi: 10.1371/journal.pone.0281345 (PMC9894498; doi:10.1371/journal.pone.0281345)
Supplement: Attachment — Submitted filename: Response to Reviewers.docx [file pone.0281345.s005.docx]

Response to Reviewer ＃1

1. Reviewer’s comment: q-RT PCR data must be confirmed by the relative protein expression in WB.

Thank you very much for your critical comment. As suggested by the reviewer, we analyzed the relative protein expression of col1, col2, and ACAN by Western blot analysis (S1 Fig).

First of all、it has been pointed out that the metabolic pathways and cellular structures change considerably when MSCs differentiate into chondrocytes, making it inappropriate to use typically used proteins as loading controls (such as GAPDH). Therefore, in this study, we used DNA content for normalizing sample quantities in Western blotting and the YWHAZ gene as a reference gene for q-RT PCR [35].

Consistent with the q-RT PCR data, undifferentiated MSCs expressed Col I but no or very low levels of ACAN and Col II. Although q-RT PCR analysis revealed that Col I, Col II, and ACAN transcript levels decreased from 3W to 5W, except for ACAN in the HA constructs, there was an increased accumulation of Col I, Col II, and ACAN proteins at 5W than 3W, indicating that these proteins continued to accumulate in the constructs, even though their mRNA expression declines during hypertrophic differentiation.

Sox-9 and MMP13 expression was confirmed by immunohistological staining (S2 Fig), because they were difficult to detect by Western blot analysis. Sox-9expression was observed in cells with fibroblast-like morphology in the cartilage at the margins of the col constructs at 3W. However, no expression was found in the well-differentiated center of the col or HA constructs, which is consistent with the fact that Sox-9 expression peaks at 3 days after MSC induction of chondrogenic differentiation, and after 3 weeks, the expression levels drop to undetectable by Western blotting (Weissenberger et al. BMC Musculoskeletal Disorders (2020) 21:109). Consistent with the q-RT PCR data, MMP-13 protein was not detected at 3W for both constructs; MMP-13 expression at 5W was confined to the marginal area in the collagen constructs, whereas it was observed in the vicinity of chondrocytes throughout the HA construct. Finally, consistent with the q-RT PCR data, the expression of Col X and OCN proteins was detected at both 3W and 5W.

Collectively, these results demonstrated that the differentiation markers of cartilage and hypertrophic cartilage are expressed in both HA and collagen constructs, although differences in mRNA and protein expression levels are observed. Raw images of Western blot are submitted as ‘S1_raw_images’.

2. Reviewer’s comment: Authors should update the bibliography.

Thank you very much for your helpful comment. We have mentioned and cited the recent papers suggested by the reviewer in Introduction (Lines 62-64) [31,32].

3. Reviewer’s comment: Alizarin Red staining in the in vitro samples must be added.

As suggested by the reviewer, we have included the data showing Alizarin Red staining in the in vitro samples (Fig 2C). As seen with Von Kossa staining, strong staining was observed limited to the outer edges in both constructs.
